# Atmospheric Pressure Dark-Current Argon Discharge Ionization with Comparable Performance to Direct Analysis in Real Time Mass Spectrometry

**DOI:** 10.5702/massspectrometry.A0075

**Published:** 2019-10-25

**Authors:** Kanako Sekimoto, Motoshi Sakakura, Hiroshi Hike, Takatomo Kawamukai, Teruhisa Shiota, Mitsuo Takayama

**Affiliations:** 1 Graduate School of Nanobioscience, Yokohama City University, 22–2 Seto, Kanazawa-ku, Yokohama 236–0027, Japan; 2 AMR Inc., 2–13–18 Nakane, Meguro-ku, Tokyo 152–0031, Japan

**Keywords:** argon, atmospheric pressure dark-current discharge, direct analysis in real time mass spectrometry, excitation, Penning ionization

## Abstract

Herein, a dark-current discharge state created by combining argon flow with a needle electrode in ambient air is described that has an ionization efficiency and mechanism comparable to those of conventional helium direct analysis in real time (DART), without requiring dopants and DART glow discharge. Using this method, polar compounds such as α-amino acids were ionized in the dark-current argon discharge *via* (de)protonation, molecular anion formation, fragmentation, (de)protonation with the attachment of oxygen, deprotonation with hydrogen loss and negative ion attachment. In contrast, nonpolar compounds (*e.g.*, *n*-alkanes) were detected as positive ions *via* hydride abstraction and oxidation. Major background ions observed were H_3_O^+^(H_2_O)*_n_*, O_2_^·+^, O_2_^·−^(H_2_O)*_n_* and CO_3_^·−^. These results indicate that the present dark-current discharge efficiently generates resonance-state argon with an internal energy of ∼14.2 eV, higher than that of the well-known metastable state (∼11.6 eV). It is therefore suggested that ionization reactions occurring there can be attributed to the Penning ionization of atmospheric H_2_O and O_2_ by resonance-state argon, analogous to helium DART.

## INTRODUCTION

Direct analysis in real time (DART) mass spectrometry was first reported by Cody *et al.* in 2005.^1)^ It is a versatile technique that operates in open air, allowing rapid, non-contact analysis of solid, liquid and gaseous materials without any pre-treatment of samples. Among other things, DART analysis can be performed directly on the surface of clothes, banknotes,^2)^ fruit,^3)^ and vegetables.^4)^ Due to its other appealing features, such as high throughput, lack of memory effect and simplicity, DART has been applied in the direct analysis of narcotics,^5)^ addictive drugs,^6)^ counterfeit drugs,^7)^ microorganisms,^8)^ warfare agents,^9)^ and pesticides,^10)^ for quality control,^11)^ in organic synthesis monitoring,^12)^ and for the surface analysis of living organisms.^13)^ In common DART, excited helium (mostly the metastable 2^3^S state, He(2^3^S)) is generated inside a ceramic flow chamber by an atmospheric pressure glow discharge using a DC voltage of 5 kV and an electric current of 3 mA. The He(2^3^S) gas is heated by passing it through a heater chamber, and then it is flowed into a sampling area through a grid electrode. The dominant positive-ion formation process is protonation, which results from the Penning ionization of atmospheric water by He(2^3^S). He(2^3^S) has an internal energy of 19.8 eV, which is higher than the ionization energy of water (12.6 eV). Penning ionization results in the generation of oxonium ions, H_3_O^+^, and its water clusters H_3_O^+^(H_2_O)*_n_*, followed by proton transfer to analytes with proton affinities greater than that of water (691 kJ mol^−1^). In negative-ion mode, analyte ionization can be attributed to proton transfer involving superoxide anion water clusters O_2_^·−^(H_2_O)*_n_*.

Although helium DART has been performed with a great amount of success as described above, helium gas is quite difficult to obtain recently, which makes it hard to sustain its use. Argon is a possible alternative gas for DART. Several research groups have investigated how argon works for DART compared to helium.^14–17)^ Excited argon stably exists in discharges (including DART glow discharge) in metastable states, such as the ^3^P_2_ and ^3^P_0_ states, with internal energies of 11.6 and 11.7 eV, respectively.^15,18)^ These energies are lower than the ionization energy of H_2_O, which results in the formation of fewer H_3_O^+^(H_2_O)*_n_* ions and give rise to quite low analyte ionization efficiency in argon DART.^15,16)^ Thus, dopant-assisted protonation based on atmospheric pressure photoionization has been used for the effective operation of argon DART.^15,17)^

Herein, a novel argon discharge ionization technique under atmospheric pressure is reported in which the analyte ionization efficiency and mechanism are comparable to those of conventional helium DART. The present discharge system was easily established by modifying the conventional DART source: (i) a needle, whose tip surface has a specific shape, is placed in the sampling area, (ii) heated ground state argon is flowed through the sampling area and (iii) low DC voltage (< 2 kV) is applied to this needle using the electrospray voltage source of the mass spectrometer. Notably, the use of dopants and a DART glow discharge are not required. The resulting discharge state in the sampling area is referred to as a ‘dark current,’ a very low electric current (0.2–1 μA) compared to the DART glow discharge. Excited state argon formed makes it possible to efficiently generate H_3_O^+^(H_2_O)*_n_* and O_2_^·−^(H_2_O)*_n_* reagent ions. These reagent ions lead to the formation of (de)protonated analytes, the abundances of which are equal to those generated in helium DART.

## EXPERIMENTAL

### Analytes

The analytes used were eight standard α-amino acids, two *n*-alkanes, anisole and benzene. Glycine (Gly), L-asparagine (Asn), L-aspartic acid (Asp), L-tyrosine (Tyr), L-tryptophan (Trp), L-alanine (Ala), L-phenylalanine (Phe) and L-methionine (Met) were purchased from Sigma-Aldrich, Japan (Tokyo, Japan). *n*-Pentadecane, *n*-heptadecane, anisole and benzene were purchased from Tokyo Chemical Industry (Tokyo, Japan). Analytes were used without further purification. The molecular properties of each analyte are summarized in Table 1.

**Table 1:** Table 1. Molecular properties of the analytes used in this work.

Analyte (M)	Chemical formula	Molecular mass	Ionization energy [eV]	Proton affinity [kJ mol^−1^]	
				Δ*H°* (M+H^+^→[M+H]^+^)	Δ*H°* ([M−H]^−^+H^+^→M)
L-Glycine	C_2_H_5_NO_2_	75	8.9	886.5	1434.0±9.2
L-Asparagine	C_4_H_8_N_2_O_3_	132	—	929.0	1385.0±9.2
L-Aspartic acid	C_4_H_7_NO_4_	133	—	908.9	—
L-Tyrosine	C_9_H_11_NO_3_	181	—	926.0	1413.0±11.0
L-Tryptophan	C_11_H_12_N_2_O_2_	204	—	948.9	—
L-Alanine	C_3_H_7_NO_2_	89	8.9	901.6	1430.0±7.9
L-Phenylalanine	C_9_H_11_NO_2_	165	—	922.9	1418.0±18.0
L-Methionine	C_5_H_11_NO_2_S	149	8.3	935.4	—
*n*-Pentadecane	C_15_H_32_	212	—	—	—
*n*-Heptadecane	C_17_H_36_	240	—	—	—
Anisole	C_7_H_8_O	108	8.2	839.6	1679.0±13.0
Benzene	C_6_H_6_	78	9.2	750.4	1678.7±2.1

The ionization energy and proton affinity values are taken from the NIST Chemistry WebBook.^35)^

### Mass spectrometry

**Figure 1:**
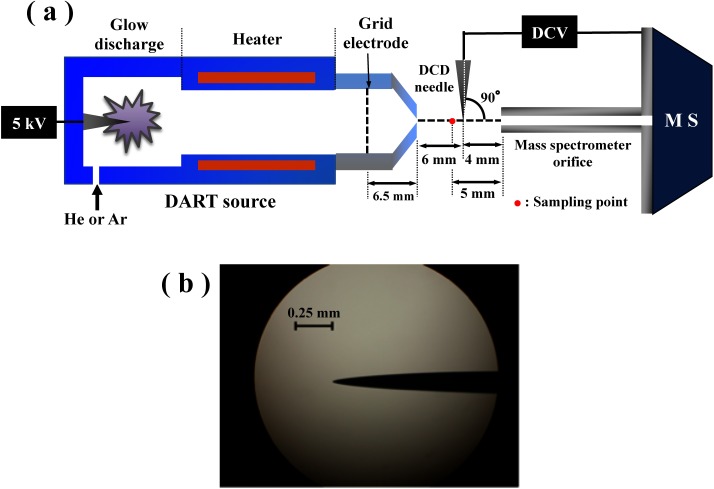
Fig. 1. (a) Schematic illustration of the present experimental setup. (b) Optical micrograph of the dark-current discharge (DCD) needle. The radius of its tip curvature is *ca.* 1 μm.

A schematic illustration of the present experimental setup is shown in Fig. 1a. Hot argon (500°C) or helium (350°C) gas was flowed through a DART-SVP source (IonSense, Saugus, MA, U.S.A.). The gas flow rates and purities were 2.0 L min^−1^ and >99.99% for both the gases. The voltages of the DART glow discharge and the exit grid electrode were −5.0 kV and 350 V, respectively. The DART source exit was directed towards the ceramic ion transfer tube of the Vapur interface or the metallic orifice of the mass spectrometer, separated by a gap of 10 mm. Vapur interface is an additional pumping system to eliminate the helium gas. The area between the DART source exit and orifice was surrounded by ambient room temperature air with a relative humidity of 40–60%. The needle used for the dark-current discharge was a 20 mm long stainless-steel pin with a diameter of 200 μm, which has a tip end radius with a curvature of *ca.* 1 μm and includes a tip end formed into a hyperboloid of revolution (Fig. 1b). This needle was placed perpendicular to the central axis of the mass spectrometer orifice, with a distance between the needle tip and the ion transfer tube/orifice of 4 mm. This needle configuration was optimized for efficient (de)protonation of analytes in low electric field. The electric field distribution established on this needle tip surface and resulting ion chemistry have been well characterized elsewhere.^19)^ DC voltages for the dark-current discharge (< ±2.0 kV) were supplied using the electrospray ionization (ESI) voltage source of the mass spectrometer. The needle counterelectrode was the DART exit grid electrode separated by a gap of 12.5 mm. Analyte desorption and ionization were accomplished by inserting a 1.5 mm ID glass tube containing the solid- or liquid-state analytes (1.0±0.2 mg) into the gas flow at a position of 5.0±0.3 mm from the mass spectrometer orifice, into which the resulting gas-phase analyte ions were introduced. Mass spectra were acquired with the following three mass spectrometers: LCQ Deca XP ion-trap (Thermo Fisher Scientific, San Jose, CA, U.S.A.), LCMS-2020 quadrupole (Shimadzu, Kyoto, Japan), or AccuTOF time-of-flight mass spectrometers (JEOL, Tokyo, Japan). All of the instruments comprised an atmospheric pressure ionization (API) interface, two differential pumping regions and analysers. However, the LCQ ion-trap and LCMS-2020 quadrupole mass spectrometers have a Vapur interface in front of the API interface. The voltages and temperatures of the orifice, lens and skimmer in the primary differential pumping region of each instrument were manually adjusted to inhibit excessive fragmentation and clustering processes. These parameters are summarized in Table 2.

**Table 2:** Table 2. Ionization modes and instrumental conditions in the first pumping stage of the (a) LCQ Deca XP ion-trap, (b) LCMS-2020 quadrupole and (c) AccuTOF time-of-flight mass spectrometers.

Ionization mode	(a) LCQ ion-trap		(b) LCMS-2020 quadrupole		(c) AccuTOF time-of-flight	
	ESI-positive/negative ion modes		ESI-positive ion mode		ESI-positive ion mode	
Instrumental conditions	Capillary (orifice) temperature	275°C	Capillary (orifice) temperature	200°C	Orifice 1 temperature	30°C
	Capillary (orifice) voltage	20 V	Capillary (orifice) voltage	0 V	Orifice 1 voltage	40–60 V
	Skimmer voltage	0 V	Desolvation line temperature	250°C	Orifice 2 voltage	5 V
	Tube lens voltage	50 V	Desolvation line voltage	0 V	Ring lens voltage	10 V

In this work, the following four sets of discharge conditions were used in terms of gas species and turn-on/off DART glow discharge and dark-current discharge (DCD): (i) Ar-DART (turn-on DART operated with Ar), (ii) Ar-DART+DCD (in which argon is flowed through turn-on DART and DCD), (iii) Ar-DCD (where argon is flowed only through turn-on DCD, but argon is heated earlier in a DART source) and (iv) He-DART (conventional DART operated with He). The details of the discharge conditions (i)–(iv) are summarized in Table 3.

**Table 3:** Table 3. The four sets of discharge conditions used in this work.

	Voltage		Gas
	DART glow discharge	Dark-current discharge (DCD)	
(i) Ar-DART	On	Off	Argon
(ii) Ar-DART+DCD	On	On	Argon
(iii) Ar-DCD	Off	On	Argon
(iv) He-DART	On	Off	Helium

## RESULTS AND DISCUSSION

### Background ions formed in the atmospheric pressure dark-current argon discharge

**Figure 2:**
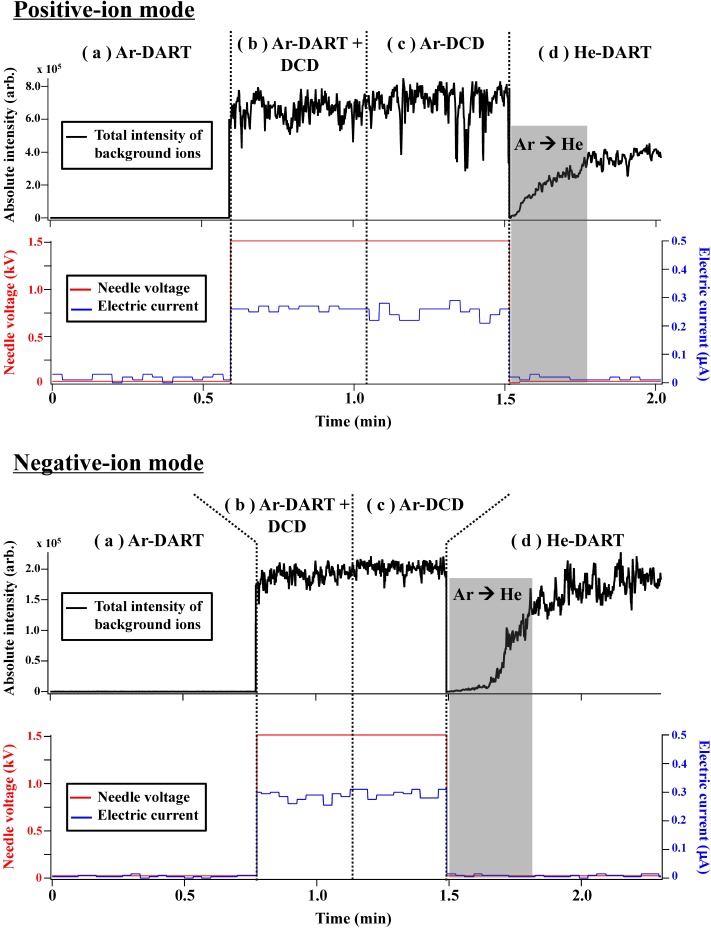
Fig. 2. Variation in the total ion intensity originating from the background air, needle voltage and resulting electric current obtained during (a) Ar-DART, (b) Ar-DART+DCD, (c) Ar-DCD and (d) He-DART. The mass spectrometer used here was the LCQ ion-trap. Gas exchange from argon to helium was conducted at ∼1.5 min. Gradual increase in intensity for He-DART (shown as shaded area) is a memory effect for gas conversion, not meaning slow time response of He-DART. Argon is heavy gas, so that it takes a little long time (∼0.25 min in this case) to completely exchange from argon to helium.

No ions were observed in background mass spectra when performing only Ar-DART (Fig. 2a). However, when the DCD needle was used, a fairly low electric current of 0.2–1 μA was recorded, and background ions were readily observed regardless of whether the DART glow discharge was turned on or not (Figs. 2b and c). The turn-on DART glow discharge was not required for forming background ions by the DCD needle, while argon flow was essentially needed. Notably, the resulting total ion intensities were very similar to those observed in the conventional He-DART technique (Fig. 2d). The turn-on DCD needle voltage of 0.2–1 μA resulted in no visible light on the needle tip or between the DART source exit and the mass spectrometer orifice. This non-self-sustaining discharge state can be described as a ‘dark current (or Townsend dark).’^20,21)^ Dark-current discharge is energetically much lower than self-sustaining discharge with visible light and electric current higher than 1 μA, which have been used in atmospheric pressure argon plasma mass spectrometry so far, *e.g.*, corona discharge used in atmospheric pressure Penning ionization (APP_e_I),^18)^ glow discharge used in Ar-DART,^14–17)^ microwave discharge used in microwave-induced plasma ionization,^22)^ and inductively coupled plasma (ICP) used in ICP mass spectrometry.^23)^ The present dark-current argon discharge (Ar-DCD) can form background ions similar to conventional He-DART.

**Figure 3:**
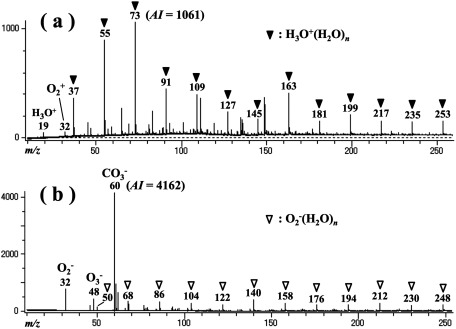
Fig. 3. (a) Positive and (b) negative background ion mass spectra obtained by Ar-DCD. The mass spectrometer used was the AccuTOF time-of-flight. AI represents the absolute intensity (arbitrary units) of a given ion.

Figure 3a shows the positive background ion mass spectrum measured by Ar-DCD. The major positive ion observed is the oxonium ion, H_3_O^+^ (*m*/*z* 19), and its water cluster, H_3_O^+^(H_2_O)*_n_* (*m*/*z* 19+18*n*). It should be noted that the molecular ion of oxygen O_2_^·+^ (*m*/*z* 32) is also present. Taking into account the ionization energy (IE) of oxygen at 12.1 eV, it is most likely that Ar-DCD efficiently generates excited state argon with an internal energy higher than those of metastable states, *e.g.*, the resonance state 5S ^3^P_1_ at an internal energy of 14.1 eV and 5S ^1^P_1_ at 14.3 eV.^24)^ It is known that these resonance states of argon (Ar*_res_) form O_2_^·+^ and H_2_O^·+^ *via* the Penning ionization of O_2_ and H_2_O with an IE of 12.6 eV (reactions 1 and 5).^24,25)^ Therefore, this suggests that H_3_O^+^(H_2_O)*_n_* is generated *via* successive reactions involving O_2_^·+^, H_2_O^·+^ and H_2_O (reactions 2–4, 6 and 7).
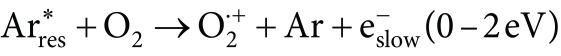
(1)
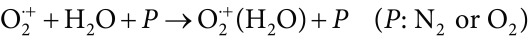
(2)
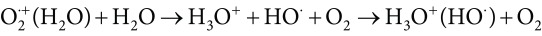
(3)
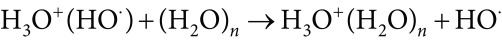
(4)
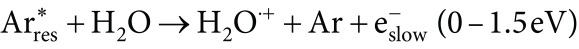
(5)
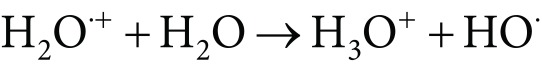
(6)
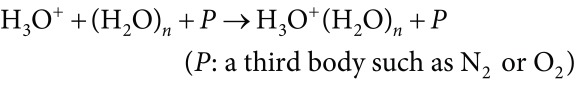
(7)

These reaction sequences also bring about the formation of hydroxyl radicals, HO^·^, and slow electrons, e^−^_slow_, with low kinetic energies of 0–2 eV. The formation mechanism of excited state argon in Ar-DCD is discussed later.

The negative core ion of the most dominant water cluster is superoxide O_2_^·−^ (*m*/*z* 32), as shown in Fig. 3b. The O_2_^·−^ ions are formed *via* the attachment of thermal electrons that have kinetic energy at ambient temperature, *i.e.*, e^−^_slow_ (∼0 eV) to O_2_ (reaction 8).
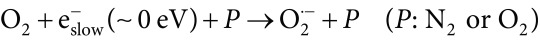
(8)

The formation of thermal electrons can be attributed to Penning ionization (reactions 1 and 5). The negative ion mass spectrum also shows a predominantly high ion peak for CO_3_^·−^ (*m*/*z* 60). This indicates that ozone, O_3_, is abundant in the discharge area. According to the difference in the electron affinities of O_2_ (0.5 eV) and O_3_ (2.1 eV),^26)^ charge transfer from O_2_^·−^ to O_3_ (reaction 9) easily occurs, and the resulting O_3_^·−^ ions formed react with CO_2_ in ambient air to generate CO_3_^·−^ (reaction 10).^27)^
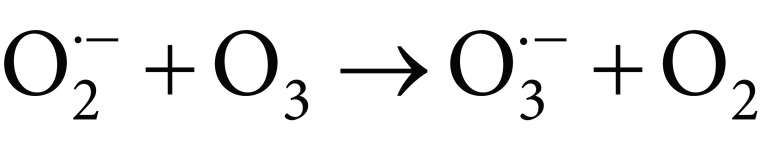
(9)
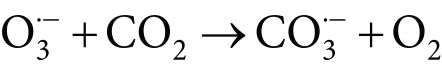
(10)

Ozone, O_3_, can be produced *via* the collision of Ar*_res_ with O_2_ to form an O atom in a ground-level triplet state, O(^3^P) (reaction 11), and the subsequent combination of O(^3^P) with O_2_ (reaction 12).
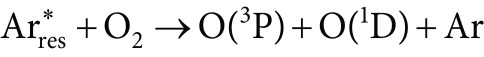
(11)
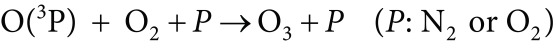
(12)

### Analyte ionization in Ar-DCD

**Figure 4:**
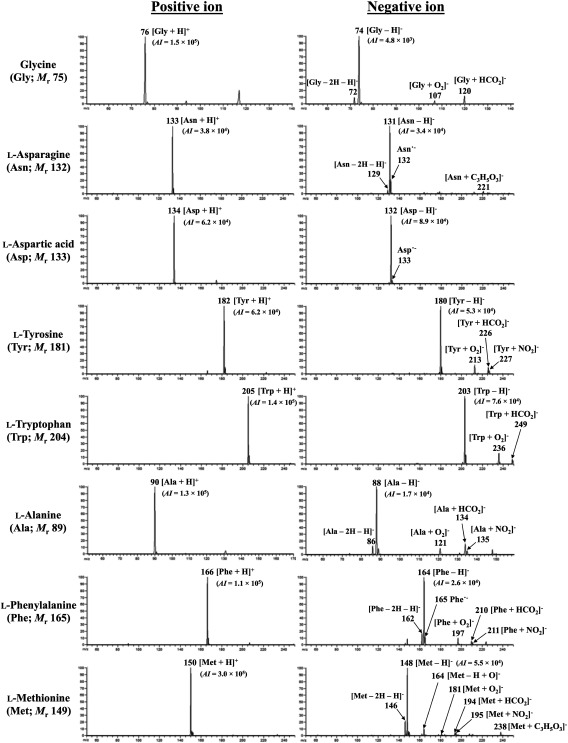
Fig. 4. Positive- and negative-ion Ar-DCD mass spectra of the eight α-amino acids obtained by the LCQ ion-trap mass spectrometer. AI represents the absolute intensity (arbitrary units) of a given ion. The ion intensities observed here are summarized in Table 4.

**Figure 5:**
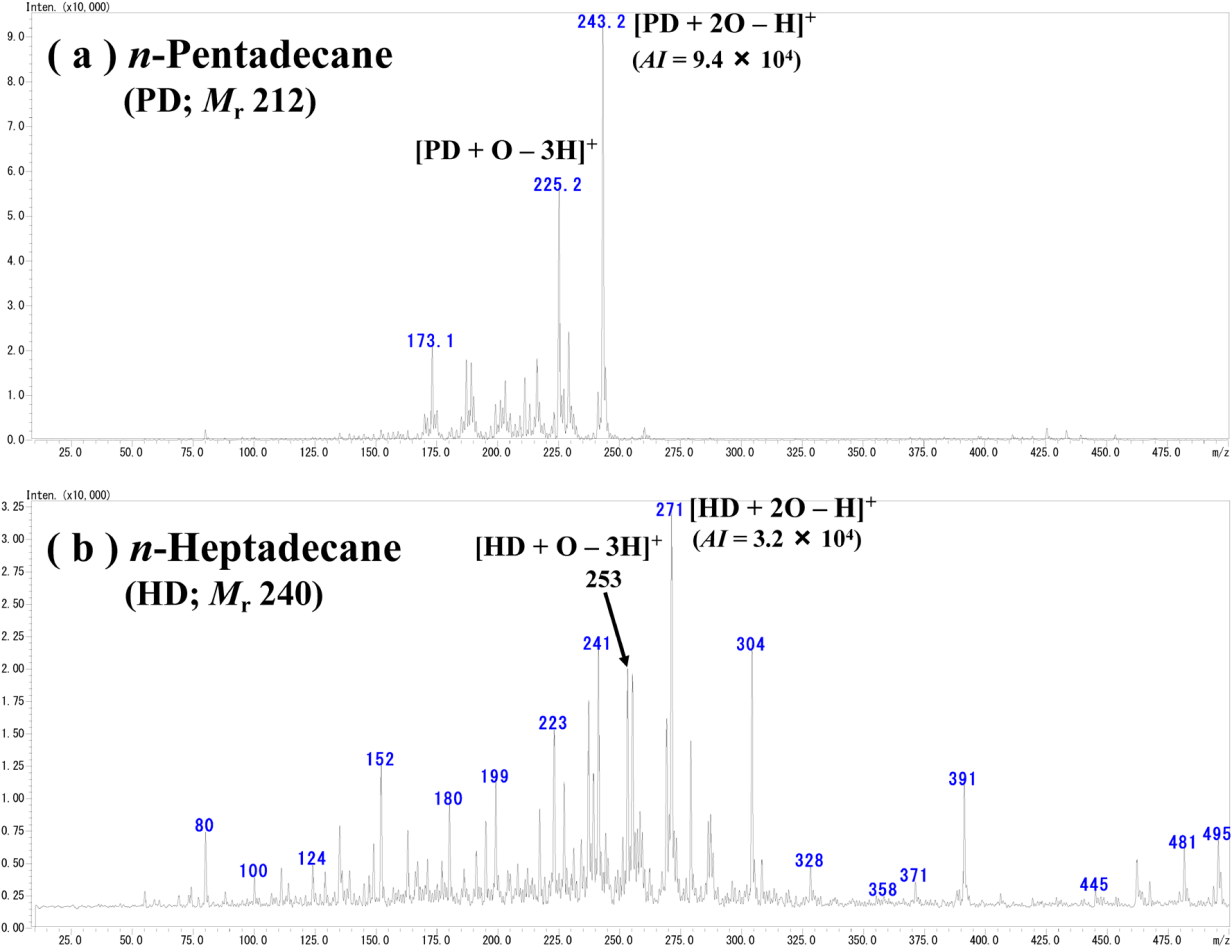
Fig. 5. Positive-ion Ar-DCD mass spectra of (a) *n*-pentadecane and (b) *n*-heptadecane obtained by the LCMS-2020 quadrupole mass spectrometer. AI represents the absolute intensity (arbitrary units) of a given ion.

Ar-DCD resulted in analyte ionization efficiencies and characteristics comparable to those of conventional He-DART for polar and nonpolar compounds. Figure 4 shows the mass spectra of eight α-amino acids (A) positively and negatively ionized in Ar-DCD. (De)protonated molecules [A±H]^±^ were dominantly observed for all of the amino acids, and the mass spectral patterns were nearly identical to those observed using the He-DART technique.^28)^ The other ion species detected were molecular anions A^·−^, oxygenated (de)protonated molecules [A±H+*n*O]^±^, dehydrogenated deprotonated molecules [A−2H−H]^−^, fragment ions [A±H−F]^±^ (F: neutral fragment) and negative ion adducts [A+R]^−^ (R^−^: negative background ions such as O_2_^·−^, HCO_2_^−^, NO_2_^−^ and deprotonated lactic acid C_3_H_5_O_3_^−^), as shown in Fig. 4 and Table 4c. Figure 5 shows the positive-ion Ar-DCD mass spectra of *n*-pentadecane and *n*-heptadecane. Both alkanes (Alk) were detected as [Alk+O−3H]^+^ (*m*/*z* Alk +13) and their monohydrates [Alk+2O−H]^+^ (*m*/*z* Alk +31), and their spectra were found to be analogous to those observed using the He-DART technique.^29,30)^

**Table 4:** Table 4. Ion species and intensities observed in the (a) Ar-DART, (b) Ar-DART+DCD, (c) Ar-DCD and (d) He-DART mass spectra of the eight α-amino acids, two *n*-alkanes and anisole.

Ion-mode	Mass spectrometer	Analyte	Ion species	*m*/*z*	Relative intensity [%] (absolute intensity [arb.])			
					(a) Ar-DART	(b) Ar-DART+DCD	(c) Ar-DCD	(d) He-DART
Positive	LCQ ion-trap	Gly	[Gly+H]^+^	76	N.D	100 (9.4×10^4^)	100 (1.5×10^5^)	100 (6.0×10^4^)
		Asn	[Asn+H]^+^	133	N.D	100 (2.6×10^4^)	100 (3.8×10^4^)	100 (1.0×10^4^)
		Asp	[Asp+H]^+^	134	N.D	100 (1.3×10^5^)	100 (6.2×10^4^)	100 (1.5×10^4^)
			[Asp+H−H_2_O]^+^	116	N.D	0.7	N.D.	N.D.
		Tyr	[Tyr+H]^+^	182	N.D	100 (2.7×10^4^)	100 (6.2×10^4^)	100 (4.1×10^4^)
			[Tyr+H−CO_2_]^+^	138	N.D	3.5	N.D	1.1
			[Tyr+H+O]^+^	198	N.D	7.2	N.D	0.5
		Trp	[Trp+H]^+^	205	N.D	100 (5.8×10^4^)	100 (1.4×10^5^)	100 (4.9×10^4^)
			[Trp+H+O]^+^	221	N.D	8.7	N.D.	2.3
			[Trp+H+2O]^+^	237	N.D	N.D	N.D	0.7
		Ala	[Ala+H]^+^	90	N.D	100 (1.6×10^5^)	100 (1.3×10^5^)	100 (1.6×10^4^)
		Phe	[Phe+H]^+^	166	N.D	100 (1.6×10^5^)	100 (1.1×10^5^)	100 (5.8×10^4^)
		Met	[Met+H]^+^	150	N.D	100 (2.5×10^5^)	100 (3.0×10^5^)	100 (4.4×10^4^)
	LCMS-2020 quadrupole	*n*-Pentadecane (PD)	[PD+O−3H]^+^	225	N.D	65	57.9	60.5
			[PD+2O−H]^+^	243	N.D	100 (1.2×10^5^)	100 (9.4×10^4^)	100 (8.6×10^4^)
		*n*-Heptadecane (HD)	[HD+O−3H]^+^	253	N.D	61.4	59.4	78.6
			[HD+2O−H]^+^	271	N.D	100 (7.0×10^4^)	100 (3.2×10^4^)	100 (5.6×10^5^)
		Anisole (AS)	AS^·+^	108	100 (4.2×10^5^)	100 (5.5×10^5^)	100 (4.7×10^5^)	100 (4.7×10^5^)
			[AS+H]^+^	109	9.7 ^a^	30.1 ^a^	57.6 ^a^	47.4 ^a^
Negative	LCQ ion-trap	Gly	[Gly−H]^−^	74	N.D	100 (1.2×10^4^)	100 (4.8×10^3^)	100 (2.6×10^3^)
			[Gly−2H−H]^−^	72	N.D	10.4	9.5	4.3
			[Gly+O_2_]^−^	107	N.D	5.4	4.6	0.7
			[Gly+HCO_2_]^−^	120	N.D	7.9	11.7	0.3
			[Gly+NO_2_]^−^	121	N.D	3.3 ^b^	N.D.	N.D.
		Asn	[Asn−H]^−^	131	N.D	100 (2.2×10^4^)	100 (3.4×10^4^)	100 (8.1×10^3^)
			Asn^·−^	132	N.D	10.6 ^c^	16.0 ^c^	18.7 ^c^
			[Asn−2H−H]^−^	129	N.D	18	5.9	4.6
			[Asn+C_3_H_5_O_3_]^−^	221	N.D	4.1	3.9	2.7
		Asp	[Asp−H]^−^	132	N.D	100 (2.0×10^4^)	100 (8.9×10^4^)	100 (2.3×10^4^)
			Asp^·−^	133	N.D	2.5 ^c^	0.5 ^c^	3.7 ^c^
			[Asp−H−NH_3_]^−^	115	N.D	0.9	N.D.	1.1
		Tyr	[Tyr−H]^−^	180	N.D	100 (2.5×10^4^)	100 (5.3×10^4^)	100 (2.3×10^4^)
			[Tyr+O_2_]^−^	213	N.D	13.7	13.7	3.8
			[Tyr+HCO_2_]^−^	226	N.D	16.7	9.7	9.7
			[Tyr+NO_2_]^−^	227	N.D	4.2 ^b^	3.9 ^b^	3.8 ^b^
			[Tyr−H+O]^−^	196	N.D	4.7	N.D.	2.6
		Trp	[Trp−H]^−^	203	N.D	100 (5.2×10^4^)	100 (7.6×10^4^)	100 (2.1×10^4^)
			[Trp+O_2_]^−^	236	N.D	16.3	16	5.8
			[Trp+HCO_2_]^−^	249	N.D	9.4	6.8	6
			[Trp−H+O]^−^	219	N.D	11.4	N.D.	7
		Ala	[Ala−H]^−^	88	N.D	100 (7.9×10^3^)	100 (1.7×10^4^)	100 (4.9×10^3^)
			[Ala−2H−H]^−^	86	N.D	16.3	13.1	5.7
			[Ala+O_2_]^−^	121	N.D	6.7	8.9	N.D.
			[Ala+HCO_2_]^−^	134	N.D	14.7	15.8	6.8
			[Ala+NO_2_]^−^	135	N.D	3.8 ^b^	4.9 ^b^	N.D.
		Phe	[Phe−H]^−^	164	N.D	100 (3.2×10^4^)	100 (2.6×10^4^)	100 (2.4×10^4^)
			Phe^·−^	165	N.D	0.6 ^c^	1.4 ^c^	2.0 ^c^
			[Phe−2H−H]^−^	162	N.D	19.5	17.1	9.5
			[Phe+O_2_]^−^	197	N.D	15	9.7	5.1
			[Phe+HCO_2_]^−^	210	N.D	6.7	5.3	5.1
			[Phe+NO_2_]^−^	211	N.D	4.8 ^b^	2.9 ^b^	3.4 ^b^
			[Phe+C_3_H_5_O_3_]^−^	244	N.D	N.D.	N.D.	6.5
			[Phe−H+O]^−^	180	N.D	N.D.	N.D.	1.1
		Met	[Met−H]^−^	148	N.D	100 (3.7×10^4^)	100 (5.5×10^4^)	100 (1.8×10^4^)
			[Met−2H−H]^−^	146	N.D	18.6	22	11.7
			[Met+O_2_]^−^	181	N.D	7.6	2.7	1.2
			[Met+HCO_2_]^−^	194	N.D	4.7	3.6	1.5
			[Met+NO_2_]^−^	195	N.D	2.7 ^b^	4.6 ^b^	1.1 ^b^
			[Met+C_3_H_5_O_3_]^−^	238	N.D	3.8	5.4	2.8
			[Met−H+O]^−^	164	N.D	16.3	10.3	3.1

^a^ Relative intensity is subtracted contribution of AS^·+^ having one isotope of ^13^C, ^2^H, or ^17^O. ^b^ Relative intensity is subtracted contribution of [Analyte+HCO_2_]^−^ having one isotope of ^13^C, ^2^H, ^17^O, ^15^N, or ^33^S. ^c^ Relative intensity is subtracted contribution of [Analyte−H]^−^ having one isotope of ^13^C, ^2^H, ^17^O, or ^15^N.

An interesting ionization characteristic of Ar-DCD is the high efficiency of protonation, comparable to that of the He-DART technique. To examine how protonation occurs in Ar-DCD, anisole was measured using the four sets of discharge conditions. As anisole has a relatively low ionization energy of 8.2 eV, it can be ionized to a molecular ion by Ar-DART alone *via* the Penning ionization of metastable argon Ar*_meta_ (reaction 13)^15)^:
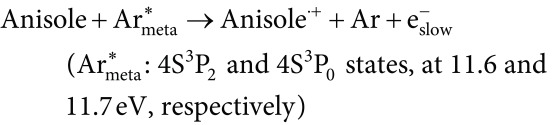
(13)

Ar-DART also forms protonated molecules with ∼10% intensity of the molecular ion (Table 4), which is consistent with what has been previously shown in the literature.^15)^ This is most likely due to hydrogen atom transfer from the neutral anisole molecule to the molecular ion (reaction 14)^18)^:
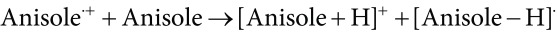
(14)

When the DCD is turned on, the relative intensity of the protonated molecule to the molecular ion increases: 28 and 58% for Ar-DART+DCD and Ar-DCD, respectively (Table 4). The 58% intensity is even higher than that observed in the He-DART technique (47%, as shown in Table 4), and these results suggest that Ar-DCD has a specific protonation pathway, other than those shown in reactions (13) and (14).

Taking into account the background ions in Ar-DCD described earlier, it is thought that the analyte ionization mechanism is the same as that observed in the He-DART technique.^28)^ The proton transfer reactions between amino acids (A) and background ions H_3_O^+^(H_2_O)*_n_* (reaction 15) and O_2_^·−^ (reaction 16) can be attributed to a (de)protonation process and possibly resonant capture of slow electrons with kinetic energies of 1–1.5 eV by A (reaction 17).
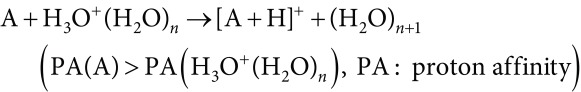
(15)
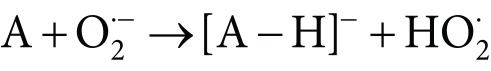
(16)
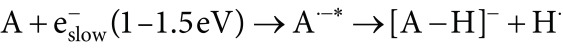
(17)

As proton affinities of neutral or deprotonated amino acids (shown in Table 1) are sufficiently higher or lower than that of H_2_O (691 kJ mol^−1^) or O_2_^·−^ (1476.9±3.0 kJ mol^−1^), reactions 15 and 17 exothermically proceed at rates close to the capture collision rates. The absolute intensities of [A±H]^±^ observed in Ar-DCD were 1.1–8.1 times higher than those in He-DART (see Table 4c and d). This result may suggest that the abundance of Ar*_res_ is higher than that of He(2^3^S) in the analyte ionization area, and resulting in higher efficiency of the background ion formation and analyte (de)protonation in Ar-DCD than He-DART. Its main reason is likely that helium with low mass of 4 can disperse more easily compared to argon with mass of 40.

Fragmentation occurs when the resulting (de)protonated molecules [A±H]^±^ have excess energy. If reaction (16) involves a third body, such as N_2_ and O_2_, then a negative ion adduct [A+O_2_]^·−^ is formed (reaction 18).
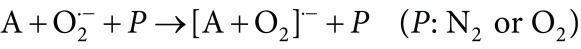
(18)

A molecular anion A^·−^ is formed *via* the resonant capture of thermal electrons by an analyte molecule (reaction 19).
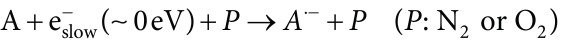
(19)

The oxidation process, in the form of both oxygen attachment and hydrogen loss processes, involves mainly HO^·^ hydroxyl radicals. A more detailed explanation of these ionization reactions can be found elsewhere.^28)^

*n*-Alkanes (Alk) can be ionized to [Alk+O−3H]^+^ and [Alk+2O−H]^+^ ions *via* hydride abstraction and oxidation processes. These two ions have been significantly observed in a helium plasma jet, where a large amount of HO^·^and O_3_ are formed.^29)^ Usmanov *et al.* recently reported the involvement of O_3_ in the formation of [Alk+O−3H]^+^ in low-pressure oxygen or air plasma,^31)^ which is consistent with the results obtained here.

An interesting feature of analyte ionization by Ar-DART+DCD is that the formation of oxygenated and/or dehydrogenated (de)protonated molecules, *i.e.*, [A±H+*n*O]^±^, [A−2H−H]^−^, [Alk+O−3H]^+^, and [Alk+2O−H]^+^, are promoted compared to Ar-DCD alone. This phenomenon indicates that the addition of Ar-DART to Ar-DCD can more efficiently produce HO^·^ and O_3_ in the ionization area *via* reactions involving O_2_, H_2_O and metastable argon Ar*_meta_, formed by Ar-DART. The formation mechanism of O_3_ by Ar*_meta_ is similar to reactions 11 and 12, while the additional formation of HO^·^ is likely attributable to the dissociation reaction of H_2_O by Ar*_meta_:
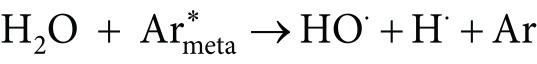
(20)

### Excitation of argon in dark-current discharge

**Figure 6:**
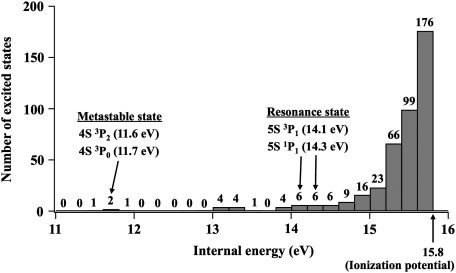
Fig. 6. Histogram of the internal energies of 425 excited states for argon.^36)^

**Figure 7:**
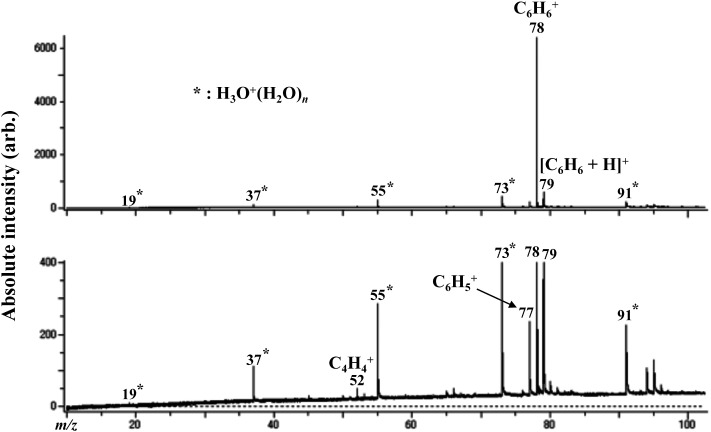
Fig. 7. Positive-ion mass spectrum obtained when argons excited by DCD directly react with benzene. The lower panel is the magnified view of the upper panel. In this case, the DCD area between the DART exit and the orifice was semi-closed and most of partial pressure inside the area consisted of argon and benzene. The mass spectrometer used here was the AccuTOF time-of-flight.

In the case of argon, it is known that there are a number of excited states up to the first ionization potential (15.8 eV), as shown in Fig. 6. Of these, the metastable states at ~11.6 eV (*e.g.*, 4S ^3^P_2_ and 4S ^3^P_0_) and the resonance states at ~14.2 eV (*e.g.*, 5S ^3^P_1_ and 5S ^1^P_1_) can occur Penning ionization.^24)^ As mentioned above, Ar-DCD efficiently generates H_3_O^+^(H_2_O)*_n_* and O_2_^·+^ (Fig. 3a). This indicates that resonance-state argon is dominantly formed in Ar-DCD. The presence of resonance-state argon was also confirmed by direct reaction of benzene with argon excited by the DCD. This reaction resulted in the production of not only molecular ion (C_6_H_6_^·+^ at *m*/*z* 78) but also several fragment ions such as C_6_H_5_^+^ (*m*/*z* 77) and C_5_H_4_^+^ (*m*/*z* 52), as shown in Fig. 7. According to the results of electron ionization (EI), these fragment ions originate from the molecular ion and their appearance energies are 14.1–14.2 eV on average.^32)^ This is consistent with energy level of resonance-state argons. The resonance state is allowed to transition to a ground state *via* the emission of UV radiation (reaction 21), however, under atmospheric pressure, this radiation is efficiently absorbed by other ground-state argon species to regenerate the resonance state (reaction 22).
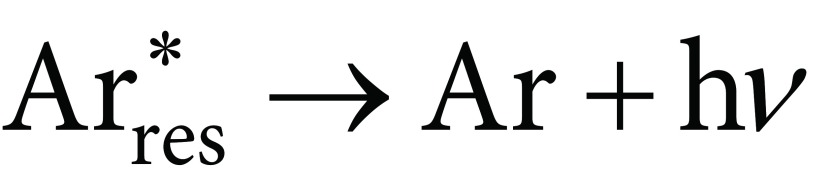
(21)
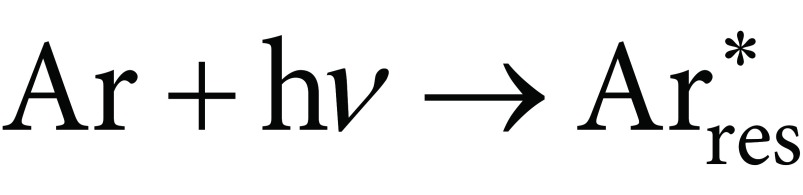
(22)

Energy corresponding to UV radiation is possessed by an argon atom for quite a long time (compared to when this energy behaves as UV radiation), which means that resonance-state argon has a long effective lifetime in ambient air.^33)^

**Figure 8:**
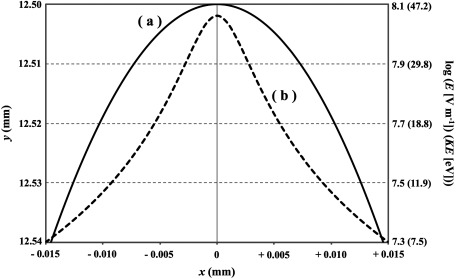
Fig. 8. (a) The hyperbola approximating the contour of the cross-section needle tip used here. (b) Logarithm with base 10 of the electric field strength on the needle tip surface (log *E*) and the corresponding electron kinetic energy (*KE*) using a discharge gap of 12.5 mm and DCD voltage of 1.5 kV as a function of the *x* coordinate of the tip surface.

The formation of resonance-state argon can be attributed to electrons that are accelerated on the DCD needle tip surface and possess a kinetic energy of over ∼14.2 eV. The kinetic energy (*KE*) of an electron is determined by the product of the electric field strength *E* and the mean free path of the electron λ_e_ (375 nm at 760 torr), *i.e.*, *KE* (eV)=*E* (Vm^−1^)×λ_e_ (m). Furthermore, the field strength depends on the local curvature of each needle tip position, in addition to the applied voltage. The dependence of the local curvature and voltage on the field strength distributions established on the needle tip surface used here have been previously examined.^19,29)^ Figure 8a shows the coordinates (*x*, *y*) of the hyperbola approximating the contour of the cross-section DCD needle tip used here. The needle tip apex, with a discharge gap of 12.5 mm, is represented by (*x*, *y*)=(0, 12.50). Figure 8b shows the logarithm of the electric field strength (log *E*) on the needle tip surface and the corresponding electron *KE*, using a discharge gap of 12.5 mm and DCD voltage of 1.5 kV. The electrons accelerated on the needle tip surface *x*=0−7.5 μm have kinetic energies ranging from 14.2 to 43.2 eV (log *E*=7.58−8.06). These electrons collide with Ar atoms flowing in the vicinity of the DCD needle tip surface though the DART source exit, resulting in the generation of resonance-state argon.

Electrons with kinetic energies of higher than ∼16 eV can form high-Rydberg state argon with an internal energy of ∼15.6 eV,^34,35)^ as well as argon ions (Ar^+^). High-Rydberg argon easily converts to dimer ions (Ar_2_^+^) upon the loss of electrons.^35)^ However, neither Ar^+^ nor Ar_2_^+^ were detected in the positive background ion mass spectrum (Fig. 3a). This may indicate that in the present discharge conditions, high-Rydberg state atoms and argon-related ions are not formed efficiently or efficient lifetimes of them are too short to detect by the mass spectrometer.

**Figure 9:**
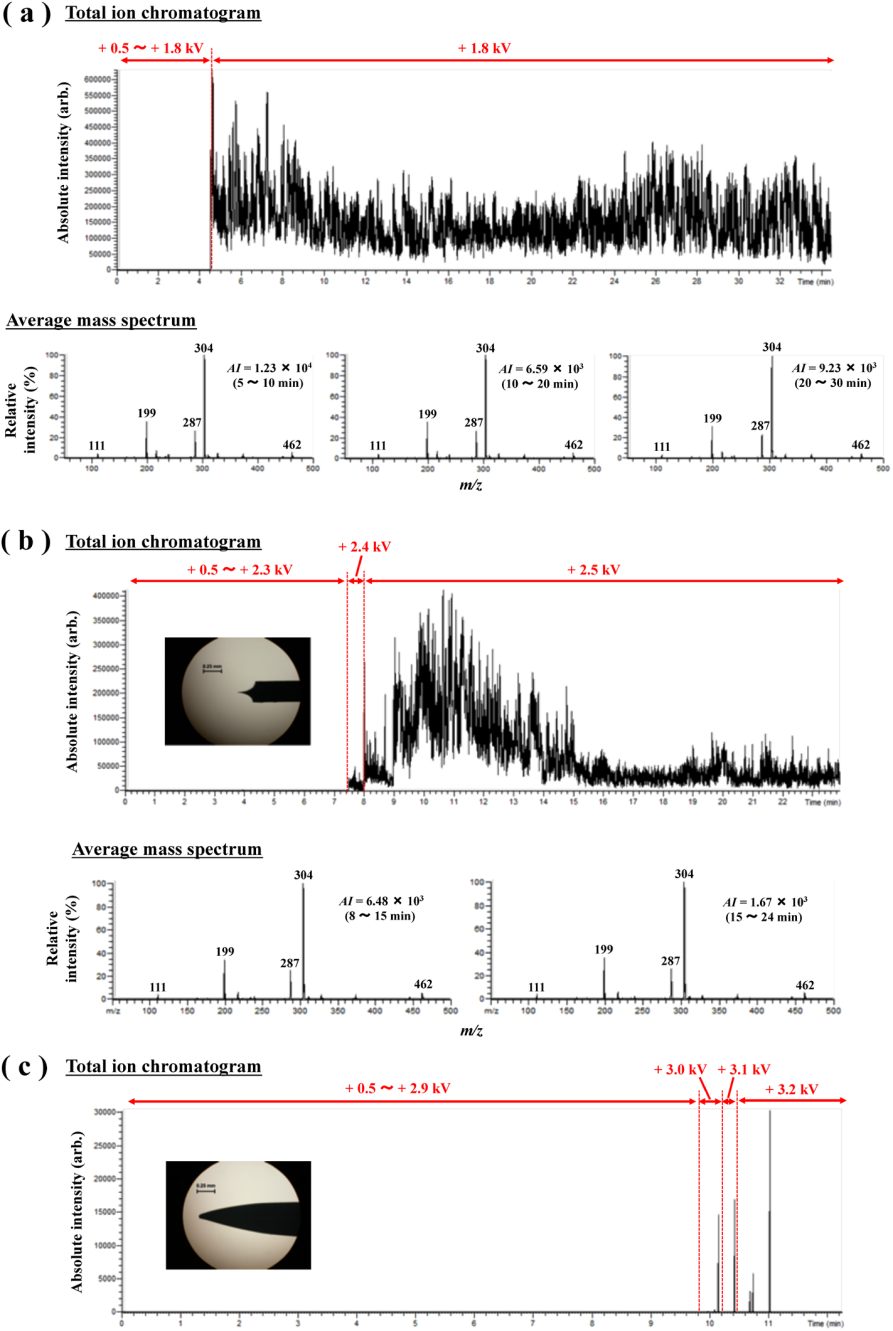
Fig. 9. Total ion chromatogram and average mass spectra of positive background ions obtained using (a) the present DCD needle (Fig. 1b), (b) a DCD needle which has a tip end radius with a curvature of less than 1 μm and includes a tip end formed into a reversed curved surface, and (c) a DCD needle, which includes a tip end formed into a hyperboloid of revolution, but has a tip end radius with a curvature of more than 30 μm. The mass spectrometer used here was the LCQ ion-trap. AI represents the absolute intensity (arbitrary units) of the base peak in a given mass spectrum.

The needle tip shape plays a crucial role in enhancing and sustaining the Ar-DCD ionization efficiency. When DCD is created by the present needle, which has a tip end radius with a curvature of *ca.* 1 μm and includes a tip end formed into a hyperboloid of revolution (Fig. 1b), the ion intensities remain constant even after a time lapse of 30 min (+1.8 kV in Fig. 9a). However, if a needle, including a tip end formed into a reversed curved surface, is used, the ion intensities significantly reduce right after the application of the DCD voltage (+2.5 kV in Fig. 9b). This is because the tip end radius of the curvature is too small, and hence the shape of the tip end surface changes with the passage of time due to degradation by thermal melting and plasma corrosion, meaning that the electric field established on the tip end surface cannot be kept constant. In contrast, if a needle with a larger radius of curvature (> 30 μm) is used, ions are not detected in the DCD state (+0.5~+2.9 kV in Fig. 9c). If the tip end radius of curvature is too large, and hence in the DCD state, it is impossible to ensure a region of the tip end position at which sufficient amounts of high-energy electrons and resonance-state argon are formed. Only when breakdown discharge involving an emission phenomenon occur beyond the dark-current state, ions are detected with a spike shape (+3.0~+3.2 kV in Fig. 9c). Therefore, the Ar-DCD needle has the following conditions: (i) the tip end has a radius of curvature that results in the generation of a sufficient number of high-energy electrons, a sufficient amount of resonance-state argon, and reagent ions H_3_O^+^(H_2_O)*_n_* and O_2_^·−^(H_2_O)*_n_*, and (ii) the tip shape must be kept constant during the application of voltage.

Finally, we should note the following interesting findings: Discharge power level of the dark-current state is significantly lower than breakdown state observed in Ar-DART. However, the dark-current has higher level than breakdown in terms of energies involved in ionization, indicating of more efficient formation of highly excited argons under lower discharge level. To clearly interpret these findings, further investigation using spectroscopy is needed, *e.g.*, detailed measurements of densities of various excited states under individual discharge states.

## CONCLUSION

An atmospheric pressure dark-current discharge state, created by combining argon with a needle electrode in ambient air (Ar-DCD), was found to have an ionization efficiency and mechanism comparable to those of conventional helium DART, without requiring the use of a dopant or DART glow discharge. Ar-DCD can ionize polar compounds such as α-amino acids (A) to (de)protonated molecules [A±H]^±^, molecular anions A^·−^, oxygenated (de)protonated molecules [A±H+*n*O]^±^, dehydrogenated deprotonated molecules [A−2H−H]^−^, fragment ions [A±H−F]^±^ (F: neutral fragment) and negative ion adducts [A+R]^−^ (R^−^: negative background ion). The absolute intensities of the (de)protonated molecules were found to be 1.1–8.1 times higher than those observed using the helium DART technique. In contrast, using Ar-DCD, non-polar compounds (*e.g.*, *n*-alkanes; Alk) were detected as [Alk+O−3H]^+^ and [Alk+2O−H]^+^ ions *via* hydride abstraction and oxidation processes. Major background ions observed using Ar-DCD were H_3_O^+^(H_2_O)*_n_*, O_2_^·+^, O_2_^·−^(H_2_O)*_n_* and CO_3_^·−^, while argon-related ions were not observed. These results indicate that Ar-DCD efficiently generates excited state argon with an internal energy higher than those of well-known metastable states (∼11.6 eV), *e.g.*, resonance states such as 5S ^3^P_1_ with an internal energy of 14.1 eV and 5S ^1^P_1_ at 14.3 eV. Therefore, this suggests that ionization reactions occurring in the Ar-DCD method can be attribute to the Penning ionization of atmospheric H_2_O and O_2_ by resonance-state argon, in a similar manner to that in the helium DART method. It was also found that the needle tip shape for DCD plays an important role in enhancing and sustaining the Ar-DCD ionization efficiency. The needle used in this work, *i.e.*, a needle that has a tip end radius with a curvature of *ca.* 1 μm and includes a tip end formed into a hyperboloid of revolution, is very suitable for this purpose. The present atmospheric pressure dark-current argon discharge ionization technique will contribute to enhancing analytical techniques based on DART.

## Notes

Mass Spectrom (Tokyo) 2019; 8(1): A0075

## Notes

Mass Spectrometry Society of Japan bestowed the MSSJ Research Award on the author. This is an invited original article of the achievement.
